# Counterintuitive method improves yields of isotopically labelled proteins expressed in flask-cultured *Escherichia coli*

**DOI:** 10.1007/s10858-025-00461-2

**Published:** 2025-03-01

**Authors:** Miguel Ángel Treviño

**Affiliations:** Instituto de Química Física Blas Cabrera, Consejo Superior de Investigaciones Científicas, Serrano 119, Madrid, Spain

**Keywords:** Protein expression, Glucose consumption, Minimal active time, Cost reduction, ^13^C labelling

## Abstract

NMR is a powerful tool for the structural and dynamic study of proteins. One of the necessary conditions for the study of these proteins is their isotopic labelling with ^15^N and ^13^C. One of the most widely used methods to obtain these labelled proteins is heterologous expression of the proteins in *E. coli* using ^13^C-D-glucose and ^15^NH_4_Cl as the sole nutrient sources. In recent years, the price of ^13^C-D-glucose has almost tripled, making it essential to develop labelling methods that are as cost effective as possible. In this work, different parameters were studied to achieve the most rational use of ^13^C-D-glucose, and an optimized method was developed to obtain labelled proteins with high labelling and low ^13^C-D-glucose consumption. Surprisingly, the optimized method is also simple and does not require monitoring of culture growth.

**Supplementary Information:**

The online version contains supplementary material available at 10.1007/s10858-025-00461-2.

## Introduction

An essential step in the study of proteins by NMR is to obtain ^13^C-, ^15^N- and/or ^2^H-labelled proteins at high concentrations to transfer magnetization between different nuclei (15N and 13C) or suppress spin diffusion (^2^H) during NMR experiments.

The most widely used method to obtain high quantities of proteins for biological studies is to produce them in heterologous systems, mainly in *E. coli*, a very well-studied system with many variants and strains. Although biofermentors allow fine, real-time control of many of the variables affecting the bacterial growth and protein expression, they are expensive, and most laboratories use simpler types of equipment, such as Erlenmeyer flasks or other flasks shaken in incubators. Using this low-tech equipment, enough protein can be obtained in most cases. For unlabelled proteins, the cells are cultured in rich medium (LB, 2YT, or other) until the cells grow at an exponential rate, usually at optical densities between 0.6 and 0.8 units. The appropriate inducer is subsequently added to the medium to initiate the expression of the selected protein.

In the case of labelled proteins, instead of the rich medium, the classical protocol uses a modification of M9 minimal medium (Miller 1972) containing ^13^C-D-glucose and ^15^NH_4_Cl as the sole carbon and nitrogen sources, while maintaining the point of induction at 0.6–0.8 OD_600_.

Over the years, many modifications have been incorporated to this basic protocol to improve the yield of labelled protein due to the high cost of the isotope sources. Therefore, two main strategies have been followed: (1) modifications to the minimal medium and growth conditions and (2) previous generation of unlabelled biomass.

The first strategy is focused on controlling variables such as the pH of the medium, which usually decreases during bacterial culture. At acidic pH, the cells stop growing and enter a stationary phase, and if the pH is lower than 4.5, further cellular growth cannot be recovered (Neidhardt et al. 1974; Sánchez-Clemente et al. 2018). To prevent this effect, buffer concentrations can be increased to the limit of solubility ((Neidhardt et al. 1974) (Cai et al. 2019)). To keep the cells in the exponential growth phase, it is necessary to maintain the bacteria in aerobic conditions, which are usually achieved with a high rate of agitation and/or the use of baffled recipients to generate a turbulent flow of medium instead of a laminar flow. Recently, Cai et al. (Cai et al. 2019) published a method in which the culture temperature was reduced. This would increase the dissolved oxygen content by approximately 10% when the culture is kept at 30 °C or approximately 20% at 25 °C compared to the usual 37 °C oxigen solubility in water (Bok et al. 2023). In addition, lower growth rates at cooler temperatures increase the number of ribosomes per cell, resulting in a higher capacity to produce the protein of interest (Marr 1991).

Concerning the second strategy, in the seminal paper by Marley et al. (Marley et al. 2001), bacteria were grown in a rich medium to 0.6 OD_600_ and subsequently centrifuged. The medium was then changed to a minimal one and the volume was diminished to reduce isotope consumption. After 1 h of adaptation for incorporation of the isotopes and generation of labelled amino acids, protein expression is induced. Variations on this protocol have been proposed. For example, Sivashanmugam et al*.* (Sivashanmugam et al. 2009) grew bacteria up to 3–5 or 5–7 OD_600_ in rich media before centrifugation and swapping to minimal medium (in this case, without volume reduction). Like Marley et al., the cells were incubated for 1–2 h in minimal medium before induction to ensure the incorporation of the isotopes into the precursors of the protein.

In both strategies, a fraction of the isotopes is consumed for the generation of biomass (strategy 1) or to ensure complete isotope incorporation and adaptation to the minimal medium (strategy 2), and it is not harnessed for labelled protein generation.

In this paper, a method that eliminates the necessity of OD_600_ monitoring and centrifugation is presented. This eliminates a stressful step for the bacteria. Additionally, conditions to minimize the nonproductive consumption of isotopes have been studied, increasing the yield of protein without sacrificing isotope incorporation, which is maintained at approximately 97–98% for ^13^C.

## Materials and methods

### Plasmids and *E. coli* strains

To analyze each variable, *E. coli* BL21star(DE3) bacteria transformed with a pET24 plasmid containing the coding sequence for the human CB1 Cannabinoid Receptor Interacting Protein 1 (CNRIP1) (UniProt Q96F85-1) and containing an Nt-(histidine)_6_ tail and a cleavage site for TEV protease were used. All experiments were carried out with this plasmid/strain except were indicated.

To confirm the optimized method, in addition to CNRIP1, a domain of PHOX2b (PHOX2b XS) (Antón et al. (2024) and the complete NEX-XF1 (UniProt O28071) were expressed in BL21star (DE3). Additionally, for the confirmatory experiments, the plasmid encoding CNRIP1 was transformed into the Turner™ (DE3) (Merck, Darmstadt, Germany), C41(DE3) and Shuffle®T7LysY (New England Biolabs, Ipswich, MA) strains.

### Culture conditions

The transformed bacteria were subsequently grown in a slightly modified M9 +  + medium (Cai et al. 2019) (Table 1) in Erlenmeyer flasks or Tunair flasks (IBI Scientific, Dubuque, IA) with capacities at least 10 times the volume of the medium to ensure enough aeration and gas exchange between the atmosphere and the medium ((Henzler and Schedel. 1991) (Takahashi and Aoyagi 2020)) To generate inocula, the transformed strain was grown in LB overnight at 37 °C. The grown inoculum was added directly to the minimal medium.

**Table 1:** Composition of modified M9 +  + minimal growth medium

Component	Concentration
LB medium	1 mL/L
Trace elements solution^1^	4.32 μM
MgSO_4_	1 mM
BME vitamins 100 × solution^2^	2.5 mL/L
Thiamine	10 mg/2L
CaCl_2_	0.2 mM
K_2_HPO_4_	109.1 mM
KH_2_PO_4_	36.7 mM
Na_2_HPO_4_	13.8 mM
D-glucose or ^13^C-D-glucose	Variable^3^
^15^NH_4_Cl	Variable^3^

^1^ Trace elements solution formulation is detailed in supplemental material. ^2^ BME vitamins 100 × solution (Merck, Darmstadt, Germany). ^3^ Different percentages of nutrients were tested. Nutrients needed for the final protocol after optimization are: D-glucose 0.2%; ^15^NH_4_Cl 0.06%. After biomass generation, additional ^13^C-D-glucose 1% and ^15^NH_4_Cl 0.3% are added

The cultures consisted of three phases: (1) biomass generation in minimal media with unlabelled glucose, (2) addition of extra glucose (labelled or unlabelled, depending on the tested conditions) for the biosynthesis of amino acids and culture for the incorporation of isotopes, and (3) induction and protein expression.

The temperatures and times in each step were 25 °C overnight (phase 1), 30 °C for a variable time (phase 2) and 20–25 °C for 24 h (phase 3), except where indicated.

Cultures for other methods, for comparison, were performed as described in their original articles (Marley et al (2001), Sivashanmugam et al (2009), Cai et al (2019)).

### Culture variables determination

Optical density was measured at 600 nm with a Nanodrop One spectrophotometer (Thermo Scientific, Waltham, MA).

The free D-glucose concentration in the media was determined with a commercial QuantiChrom glucose assay kit (Bioassay Systems, Hayward, CA), which generates blue color due to the formation of an imino bond between the aldehyde group of sugars and o-toluidine. After a centrifugation pulse of the culture to remove the bacteria, 2–5 µl of the supernatant were mixed with 50 µl of the commercial reactive, following the manufacturer’s instructions. A_630_ measurements were performed with a Nanodrop One spectrophotometer, and the D-glucose concentration was calculated by interpolating in calibration curves obtained during the same experiment.

### Expressed protein quantification

To normalize the quantity of protein produced per gram of glucose, volumes corresponding to equivalent amounts of D-glucose added during phase 2 were collected at the endpoint of the expression (i.e., 100 μL were collected for 1% added 13C-glucose, 200 μL for 0.5% added 13C-glucose or 250 μL for 0.4% 13C-glucose. In that way (100ul*1%) = (200ul*0.5%) = (250*0.4%) = 1 mg of added ^13^C-glucose in each sample). Cultures were centrifuged, and the pellets were lysed with 50 μL of bugbuster detergent (Merck, Rahway, NJ). After centrifugation, the insoluble fractions were solubilized in 50 μL of 8 M urea. Both fractions were pooled and mixed with the same volume of loading buffer for PAGE. Two to 10 μL samples were loaded in a custom gradient (4–25%) acrylamide gel containing 3.75% trichloroethanol for direct fluorescence detection of tryptophans (Kazmin et al. 2002), and the proteins were separated by PAGE. Each sample was loaded at least three times.

Bands were quantified in a ChemiDoc MP Imaging System (Bio-Rad, Hercules, CA) using the free stain option and analyzed with ImageLab software.

### Protein purification

For mass spectrometry experiments, bacteria from 5 ml of growth cultures were centrifuged and resuspended in 1 mL of 50 mM potassium hydrogen phosphate (pH 8), 300 mM NaCl, and 10 mM imidazole with 1 μL of Halt inhibitors (Thermo Scientific, Waltham, MA). The cells were sonicated, and the lysate was centrifuged. One hundred microliters of nickel high-density beads (Agarose Bead Technologies, Torrejón de Ardoz, Spain) were added, and the mixture was loaded onto a MicroBiospin empty column (Bio-Rad, Hercules, CA). After washing with the same buffer, CNRIP1 was eluted in 400 μL of 50 mM potassium hydrogen phosphate (pH 8), 300 mM NaCl, and 500 mM imidazole. Two micrograms of TEV protease were added, and the sample was dialyzed against 1 L of 5 mM potassium dihydrogen phosphate, (pH6.8), 10 mM NaCl, and 1 mM β-mercaptoethanol.

For NMR spectroscopy, the cultures were scaled to 50 or 100 mL. Lysis was performed analogously, but the lysate supernatants were loaded in HisTrap 5 mL FF columns (Cytiva, Marlborough, MA). The eluates were dialyzed against 5 mM potassium dihydrogen phosphate (pH8), 10 mM NaCl, and simultaneously cleaved with TEV protease. The dialyzed samples were loaded in the same column and the flowthrough collected and redialyzed. The samples were then loaded onto HiTrap 1 mL SP columns (Cytiva, Marlborough, MA). The samples were prepared in 5 mM potassium dihydrogen phosphate (pH 6.8) and 10 mM NaCl.

### Mass spectrometry

The mass of the purified proteins was determined by mass spectrometry. The samples were analyzed in an HPLC 1100 Series LC System (Agilent Technologies, Palo Alto, USA) coupled to an HTC-Ultra ETD II ion trap mass spectrometer (Bruker Daltonics, Fremont, USA) with an electrospray ionization (ESI) source. The molecular masses of the proteins were calculated by deconvolution of the ESI‒MS spectra using the Thermo Finningan BIOMASSTM software (Thermo Fisher Scientific, San José, CA).

### NMR spectroscopy

1D ^1^H-spectra and ^1^H-^13^C-HSQC spectra were recorded on a Bruker Avance Neo 800 MHz (^1^H) spectrometer fitted with a cryoprobe and z-gradients. The experiments were recorded at 25 °C.

For coupled spectra, the same experiments were performed as for conventional decoupled spectra, but no ^13^C decoupling pulses were applied during acquisition.

## Results and discussion

### Biomass generation vs. available nutrients for protein expression

Previous work (Cai et al. 2019) has shown that in *E. coli* cultures at low temperatures, up to OD_600_ = 6, before induction, a high amount of protein (relative to the amount of medium used), with isotopic labelling of approximately 97% is obtained. Despite this good result, there is a percentage of the ^13^C-D-glucose that is used just to generate biomass and therefore “wasted” to improve the yield of labelled protein.

A simple way to improve this would be to combine this protocol with a first step of biomass generation in rich media, similar to other protocols, which switch to labelled media by centrifugation and produce high yields of labelled proteins. One drawback of this approach is that centrifugation steps can be stressful for the bacteria, and they recover slowly. In fact, the OD_600_ can drop in the first few moments in minimal media, and it is difficult to estimate how long the bacteria need to be grown in these media before induction to maximize expression and minimize detrimental unproductive consumption of labelled nutrients.

Therefore, it can be hypothesized that the complete consumption of unlabelled glucose in minimal media could be as efficient in terms of biomass production as using rich media but avoiding the stress of centrifugation. It has been reported that *E. coli* recover quickly from short periods of starvation with no apparent sequelae (Lempp et al. 2019). In addition, the cells adapt to grow in these minimal media from the beginning, further reducing the stress of switching from rich to poor media and minimizing the time required to incorporate labelled metabolites.

**Fig. 1:**
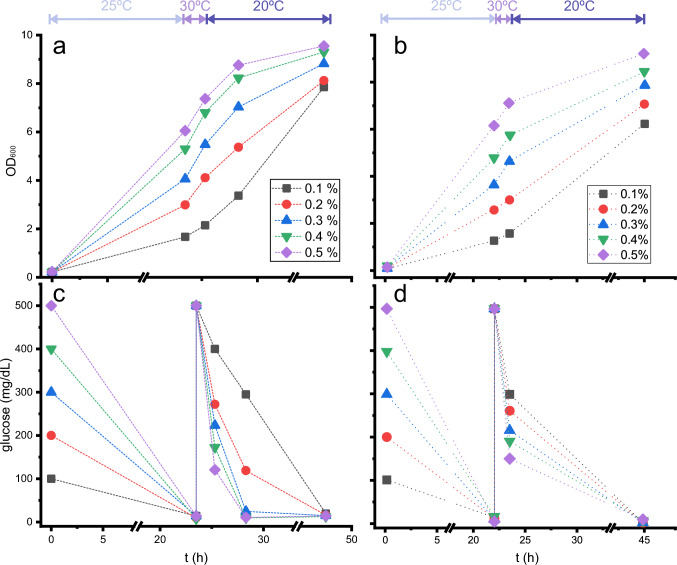
Optical density (**a**, **b**) and dissolved D-glucose (**c**, **d**) in *E. coli* cultures as a function of initial D-glucose concentration. Cultures were kept at the temperatures indicated at the top of the figure, mimicking the steps in the induced cultures, i.e., 25 °C during the ‘biomass generation step’, 30 °C after the addition of 0.5% ^12^C-D-glucose (**a**, **c**) or 0.5% ^13^C-D-glucose (**b**, **d**) and 20 °C after the induction with IPTG (‘expression step’). The dashed lines do not represent linear growth and are only added to help locate points from the same conditions

To evaluate this hypothesis, biomass production and glucose consumption were monitored under different conditions (Fig. 1a, c). Different initial D-glucose concentrations were tested. In all the samples, after 23 h of growth at 25 °C, 0.5% D-glucose was added, and the culture continued to grow at 30 °C for 1.5 h, followed by growth at 20 °C for another 24 h. The depletion of glucose after overnight growth was complete under all the conditions tested, and the growth rate recovery appeared to be complete after the addition of supplemental 0.5% D-glucose. Although it was predictable that some of this additional D-glucose would be consumed during the isotope integration step, after 1.5 h at 30 °C, the remaining nutrient content was extremely low for the cultures with high initial glucose concentrations, leaving around 0.1% D-glucose available for the protein expression step under the initial 0.5% D-glucose condition. After 3 additional hours at 20 °C, no glucose remained for the 0.3, 0.4 or 0.5% initial glucose conditions.

Differences due to switching from one isotope to the other –^12^C to ^13^C– in the “isotope integration step”, while keeping the rest of the conditions unchanged, were also tested (Fig. 1b, d). Although there is some variability during growth, as indicated by OD_600_ measurements, even before the addition of labelled glucose -probably due to the different colony used for each set of experiments-and it seems that the behaviour for ^13^C-D-glucose consumption is slightly different from that for ^12^C-D-glucose, the final global behaviour is very similar for both isotopic intakes: initial unlabelled glucose was completely consumed after 23 h at 25 ℃. When new glucose is added, ^12^C or ^13^C, the higher the initial glucose, the higher the consumption of glucose before IPTG induction and, after IPTG addition, glucose is completely consumed after 24 additional hours. Other studies indicated a small influence of ^13^C or ^12^C glucose in the metabolism of *E. coli* (Sandberg et al. 2016) and even a slight decrease of growth (usually under a 5%) using ^13^C-glucose (Xie and Zubarev 2015).

Thus, a counterintuitive result was found: it is not convenient to produce large amounts of biomass but rather to find a compromise between biomass and the consumption of labelled glucose to improve the final protein yield.

### Influence of initial glucose/labelled glucose ratio on isotope incorporation

**Fig. 2:**
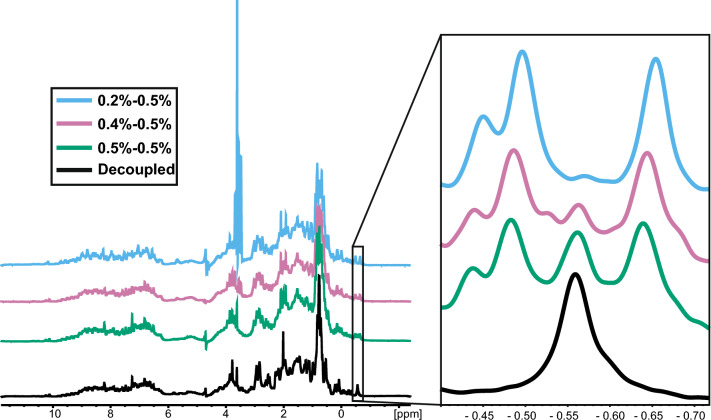
^13^C incorporation under different culture conditions measured by NMR. Spectra with or without a ^13^C decoupling pulse are shown. On the left, the full spectra are shown; on the right, the selected region is magnified. To improve clarity, the spectra are shifted vertically. For each pair of numbers naming each condition, the first of the two indicates the initial percentage of unlabelled glucose in the culture and the second indicates the percentage of 13C-glucose added after consumption of the previous one

The second variable monitored was the incorporation of ^13^C into the labelled protein. The data show that at high initial glucose, only partial incorporation into the protein was reached, but when the initial glucose was reduced from 0.5 to 0.4% and to 0.2%, the incorporation increased from 75 to 90% and 98%, respectively, as detected by NMR (Fig. 2).

**Fig. 3:**
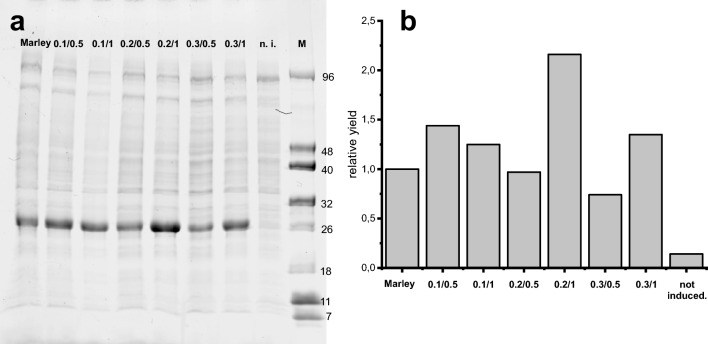
Yields of protein with different percentages of initial D-glucose and D-glucose added. For each condition, the first number indicates the initial glucose percentage in the culture and the second one the percentage of ^13^C-glucose added after consumption of the previous one. n.i.: not induced; Marley: Marley et al (2001) conditions. **a**: Fluorescence emission under UV exposure of an SDS‒PAGE gel. **b**: Bar graph representing the relative fluorescence according to the method of Marley et al. considered as a unit

These results led to the testing of other conditions—decreasing the glucose in the biomass generation step and increasing the labelled glucose added in the “isotope integration step”. The protein yield under each new condition was greater for 0.2–0.3% D-glucose in the “biomass generation step” combined with at least 1% D-glucose added in the “isotope integration step” (Fig. 3).

### “Isotope integration step” duration

**Fig. 4:**
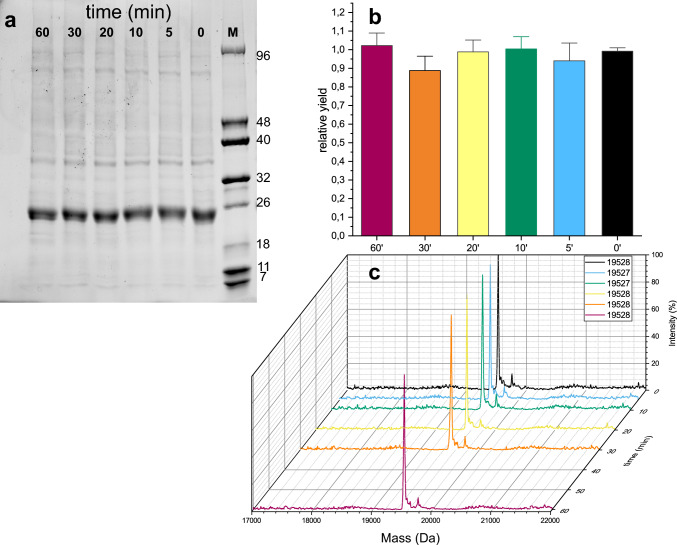
Effect of different “isotope integration” times (in minutes) on yield and ^13^C labelling. **a**: Fluorescence emission under UV exposure of an SDS‒PAGE gel. Rightmost lane are molecular weight markers. **b**: Bar graph representing the relative fluorescence according to the method of Marley et al. 2001, which was used as a unit. The data are the mean of three experiments. Error bars are for standard deviations. **c**: Mass spectra. The masses of the more intense peak for each condition are given in the square. The expected mass for CNRIP1a is 18,705 Da without labelling and 19,548 with complete 100% ^13^C incorporation. To calculate the percentage of incorporation, the formula $\% {\text{ incorporation}} = 100 \times \, \left( {{\text{experimental result }} - {\text{ theoretical mass without labelling}}} \right)/\left( {{\text{theoretical mass at }}100\% {\text{ labelling }} - {\text{ theoretical mass without labelling}}} \right)$ can be used. So, for masses of 19,527 and 19,528 Da, the percentage incorporation was 97.5 and 97.6%, respectively

Considering the data from the previous experiments, the effect of varying the time of the “isotope integration step” was tested by monitoring the protein yield and ^13^C incorporation (Fig. 4, Supplementary Fig. 1). A second counterintuitive fact emerged: there is a minimal effect of the length of this phase on ^13^C incorporation, which is always approximately 97–98% and can even be eliminated without any effect on yield of protein or ^13^C incorporation.

As the initial data indicated that for 0.5/0.5% conditions, even with a 1.5 h isotope integration step the incorporation was approximately 75%, it seems that, in any case, the relative ratio between the initial unlabelled and subsequently added labelled glucose should not exceed 20% (0.2% initial D-glucose, 1% labelled D-glucose).

### Glucose depletion vs. temperature

**Fig. 5:**
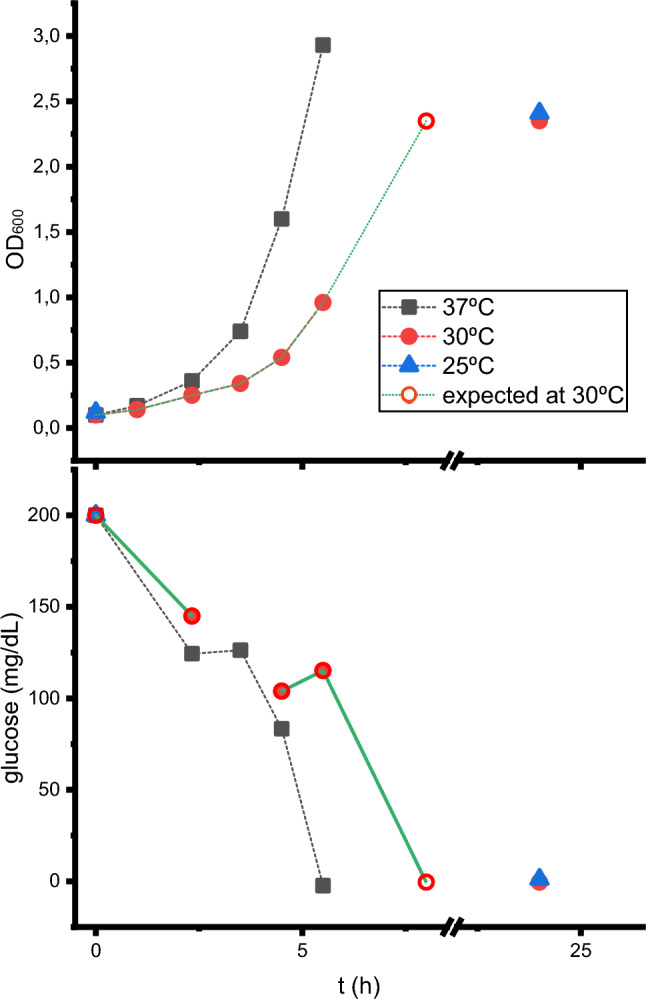
Optical density (top) and dissolved D-glucose (bottom) in *E. coli* BL21star(DE3) cultures as a function of temperature. The dashed lines do not represent linear growth and are only added to make it easier to locate symbols from the same conditions. At 30 °C, no recording was made at the likely hour of complete D-glucose consumption, so an unfilled circle was added on the basis of the exponential behavior of the curves to provide an indication of the approximate time of the event

Although all biomass generation cultures were performed at 25 °C to ensure maximum O_2_ availability to the cells, the influence of temperature on growth was monitored at 25 °C, 30 °C and 37 °C (Fig. 5). Complete glucose depletion is reached in overnight cultures at 25 °C or 30 °C, whereas at 37 °C, complete glucose depletion is achieved after approximately 5.5 h. This would allow the biomass generation step to be shortened. However, the high growth rate at 37 °C could have disadvantages, such as a lower number of ribosomes per cell (Marr 1991) or a possible microaerobic state, which could promote the accumulation of acetate (Partridge et al. 2007) and therefore inhibit cellular growth or the expression of the desired protein (Shiloach and Fass 2005). Finally, other *E. coli* strains or plasmid-strain combinations may be less efficient in nutrient consumption than those tested here and may require longer culture times. Although the total time for cultures at 25 °C or 30 °C is longer, the active time for the present method decreases from approximately 1–2 h (due to measurements of the OD_600_ until it reaches 0.6, centrifugation, resuspension in minimal medium, and addition of inductor) to approximately 5–10 min (due to the addition of labelled nutrients and inductor simultaneously) (see Table 2). Taking all these facts into consideration, for this method, it is recommended to grow the culture at 25 ℃.

**Table 2:** Time spent in different protocols

	Marley et al^1^	Cai et al^2^	Sivashanamugam^3^	This work
Overall time pre-induction	4–6 h	8–12 h	8–10 h	22 h
Induction time	4 h	20 h	20 h	22 h
Total time	8–10 h	28–32 h	28–30 h	44 h
Active time	2 h	1–1.5 h	2 h	5 min

Times are variable depending on the growth of the bacteria, except for “This work”, where the time is constant for all the different protein-strain combinations. Induction times are also variable, reflecting the times described by the authors in their original articles, but they can vary depending on toxicities, solubilities, etc. for each particular protein. Active times include OD_600_ measures (5–10 min), washes and resuspensions (10–20 min), centrifugations (20–30 min). The intermediate times between actions are not considered. ^1^Marley et al. (2001); ^2^Cai et al. (2019); ^3^Sivashanmugam et al. (2009).

Although we have not tested the possibility of starting the culture directly from fresh colonies from a plate instead of an overnight liquid preinoculum culture, as previously reported (Sivashanmugam et al. 2009), this could also diminish the total time of culture in addition to the already reduced active time.

### ^13^C-Glucose concentration optimization

**Fig. 6:**
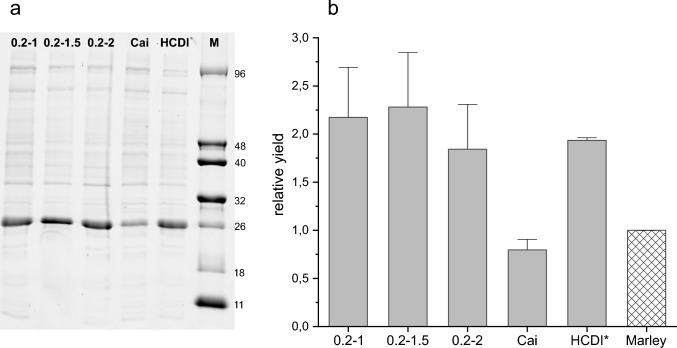
Effect of the percentage of D-glucose added in the “protein expression step” on protein yield. The pair of numbers naming each condition indicates the initial glucose concentration and that added after consumption of the first one (both in percentage); Cai indicates conditions from Cai et al, (2019) and HCDI* conditions from Sivashanmugam et al. (2009) with 3 times more NH4Cl than the original. **a**: Fluorescence emission under UV exposure of an SDS‒PAGE gel. **b**: Bar graph of relative fluorescence of the band corresponding to CNRIP1. Band intensities were referenced to the HCDI* band. For comparison, a checkered bar corresponding to the relative yield obtained using Marley et al. protocol, which was determined in previous experiments, was added to the graph. The data are from five experiments

The influence of different glucose concentrations during the expression step on yield was also investigated (Fig. 6). A slight increase in yield was detected when the glucose concentration in the expression step increased from 1 to 1.5%, but the yield decreased at higher percentages. In any case, the increment of yiels obtained adding 1.5% 13C-D-glucose is within the statistical margin of error when compared with the yield obtained adding 1% ^13^C-D-glucose Thus, we have chosen 0.2% initial unlabelled + 1% added labelled as universal conditions for the method in an attempt to keep the conditions in a safe range where, given the possible variability between plasmid and strain combinations, we can ensure that we avoid concentrations too close to those that produce a significant decrease in expression/^13^C consumption ratio. Slight improvements can be achieved for individual proteins and/or strains. In any case, this method clearly improves the yield of labelled protein compared with some of the previously described protocols, and even in the least favorable case, there is a 20% increase. The tests indicate that 50 mL of culture (0.5 g of ^13^C-D-glucose) is sufficient to obtain 10–20 mg of purified labelled protein. Notably, Sivashanmugam’s protocol was modified, and 3 times more NH_4_Cl was used for this experiment. When the original amounts were used, the yield was drastically reduced (see Fig. 9 below), indicating the importance of an appropriate ratio of nutrients to obtain the maximum yield in any protocol.

The ratios of NH_4_Cl and D-glucose in this recipe were calculated to ensure that glucose was the limiting nutrient. In this way, it can be ensured that all the unlabelled glucose has been depleted in the biomass production step. Since ^13^C-labelled proteins for NMR are usually also ^15^N-labelled, ^15^NH_4_Cl must be used from the beginning because it cannot be assured that there is no remaining nitrogen that has not been consumed in the initial steps. Since ^15^NH_4_Cl is much cheaper than ^13^C-D-glucose, net savings still prevail, and it will be even more significant if we need to use D7-^13^C-D-glucose or other isotope-labelled precursors, which are even more expensive than ^13^C-D-glucose.

### Scale up influence

**Fig. 7:**
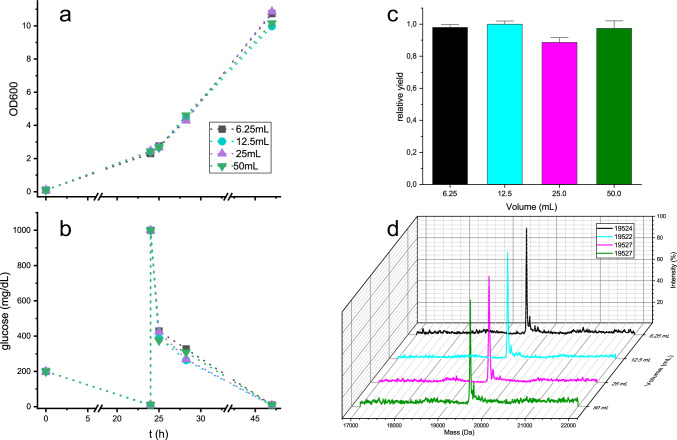
Effect of different volumes of culture for constant media volume/Erlenmeyer volume ratios. **a**. Optical densities. **b**. Free D-glucose in media. **c**. protein yield quantified for fluorescence emission after SDS-PAGE. Values are mean of three experiments. Error bars indicate standard deviations. **d**. Mass spectra. for each condition. % Incorporation for each condition were: 97.2 for 6.25 mL, 97.0 for 12.5 mL and .97.5 for 25 and 50 mL

Some of the optimizations described above were carried out in small volumes, usually 5 ml while the productions for NMR samples were carried out with 50–100 ml of media. To check if there was any effect on incorporation or growth due to the different scale, different volumes of media were tested keeping the same ratio (1/20) to the Erlenmeyer volume (Fig. 7). Although there are slight differences in OD_600_ and glucose consumption between each condition, there is no direct correlation with the culture volume. In any case, the isotope incorporation of around 97%, and similar protein yields are maintained for all the conditions, indicating that the protocol is consistent regardless of the culture volume, at least in the ranges tested. Volumes of 50–100 ml of culture provide enough labelled protein for 0.5 ml samples of approximately 0.5–1 mM.

### Universality and applicability of the method: other proteins and other strains

**Fig. 8:**
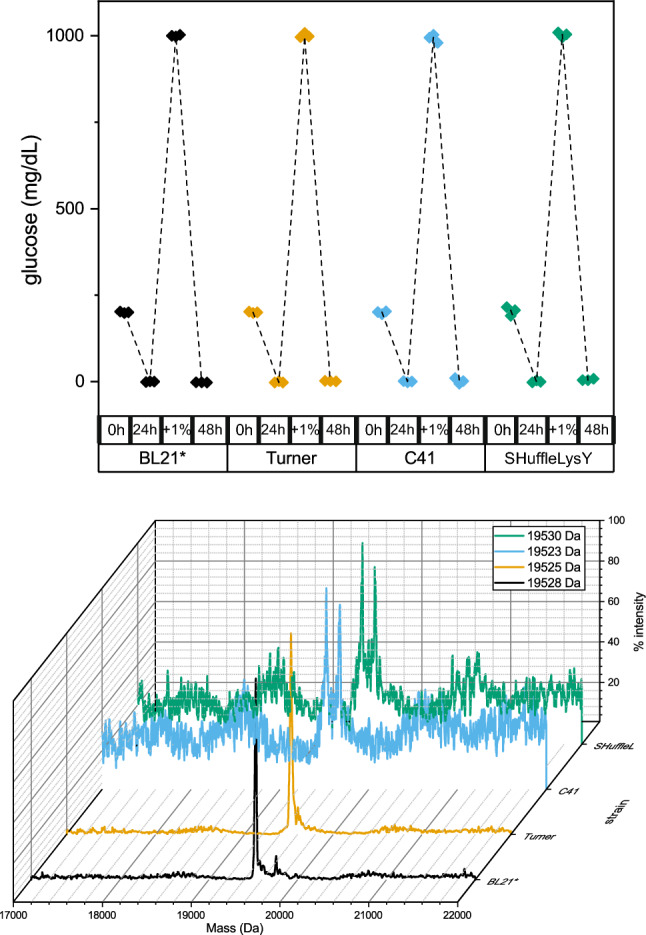
Effect of the *E. coli* strain on glucose consumption and protein expression. Top: dissolved glucose at different culture times for 3 different *E. coli* strains. Bottom: mass spectra of CNRIP1a expressed in different strains with the method described in this paper. The mass of the more intense peak for each condition is given in the square. To calculate the percentage of incorporation the formula $\% {\text{ incorporation}} = 100 \times \, \left( {{\text{experimental result}} - {\text{theoretical mass without labelling}}} \right)/\left( {{\text{theoretical mass at }}100\% {\text{ labelling}} - {\text{theoretical mass without labelling}}} \right)$ can be used. So, for masses of 19,523, 19,525, 19,528, and 19,530 Da, percent incorporation was 96.9%, 97.3%, 97.6%, and 97.9% respectively. The second intense peak detected for some strains corresponded to the double ionized DNaseI added during purification

**Fig. 9:**
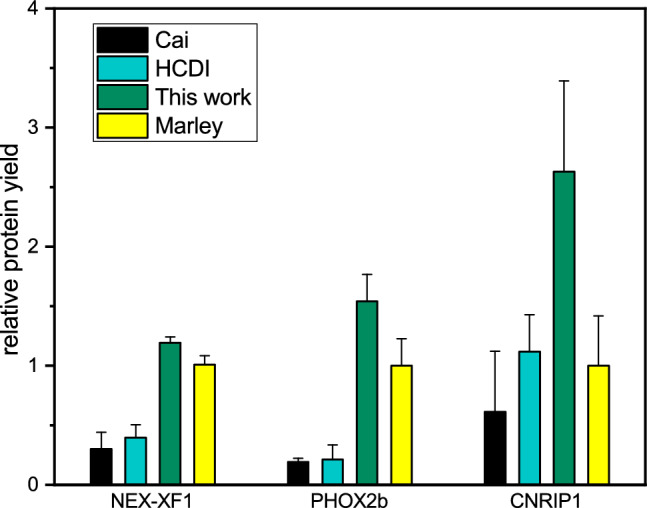
Relative protein yield for three different proteins using different protocols. Each protein expression is referenced to the expression obtained using Marley’s protocol, which is considered a unit. Error bars indicate standard deviations

Finally, two experiments were carried out to test whether this method could be used universally, regardless of the strain or protein expressed. To check for strain independence, CNRP1 was expressed in the BL21star(DE3), Turner™(DE3), C41(DE3), and Shuffle® T7 LysY strains, and ^13^C incorporation was determined by mass spectrometry. No significant differences in glucose uptake or protein expression were found between these strains and the ^13^C incorporation was almost identical (97.6%, 97.4%, 97.9% and 96.9%, respectively) (Fig. 8). To determine the variations of yield in the expression of different proteins depending of the protocol used, the method was tested on the proteins CNRIP1, PHOX2b XS and NEX XF1 (Fig. 9). In all of them, a higher quantity of protein was obtained with the method described here than with the other protocols. Although, the ratios between the different methods are not exactly the same for the three proteins, the yield per gram of glucose is higher with this method for the three proteins tested.

## Conclusions

**Fig. 10:**
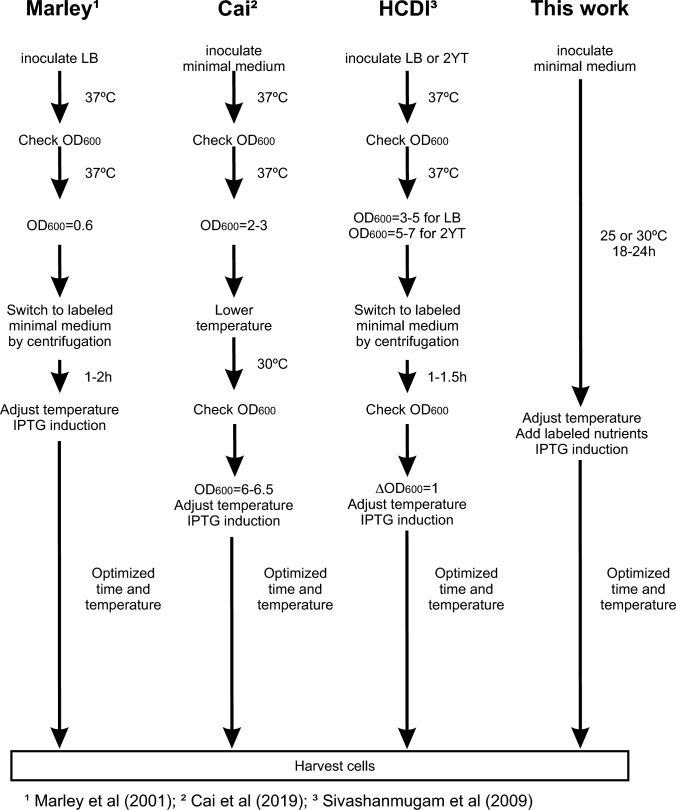
Outline of some protein expression methods compared with the one described here. Different OD_600_ measurements, centrifugations and manipulations may be required for these methods, increasing the required active time

After an exhaustive optimization of different steps (Table 3), a new method for the production of ^13^C-labelled proteins has been developed (a detailed protocol is provided in the supplemental material), which minimizes the active time commitment (Fig. 10, Table 2) and optimizes the consumption of labelled nutrients.

**Table 3:** Variables tested and optimized

Variable	Range of optimization	Final condition
Biomass-nutrients available	0.1–0.5% initial glucose	0.2%
Ratio unlabelled/labelled glucose	0.1%/0.5% to 0.3%/1%	0.2%/1%
Isotope integration step	60 min-0 min	0 min (step not neccessary)
Glucose depletion depending on temperature	0–24 h, 25–37 ℃	20–24 h at 25 ℃
^13^C-glucose concentration	1–2% labelled glucose for 0.2% initial unlabelled glucose	1%
Scale up	6.25–50 mL	No influence
Strain influence	4 different *E. coli* strains tested	Valid for all of them
Different proteins	3 different proteins tested	All show higher expression with this protocol

An initial culture step at 25–30 °C in minimal medium allows biomass to be generated while avoiding centrifugation. No “isotope integration step” is required, and simply adjusting the temperature for the optimal protein expression before adding labelled nutrients and the inductor is enough to obtain maximum protein yields with high ^13^C incorporation.

Although the total time required for the protocol described here is higher than for other protocols, there is a huge advantage in the reduced active time required. In addition, with other methods, it is difficult to calculate the exact points at which the required optical densities are reached, with the risk of exceeding the optimum density. Furthermore, depending on the protein-strain combination, some of the intermediate points where intervention is required may be reached when the working day has exceeded its usual limits.

This protocol, with minimal modifications, could be used to generate other labels, such as selenomethionine for X-ray crystallography and for the selective labelling of different amino acid positions using 1,3-^13^C-glycerol or 2-^13^C-glycerol or other precursors as carbon sources. As well as for triple (^2^H, ^15^N, ^13^C) labelled proteins.

In summary, two unexpected and counterintuitive findings, the limited biomass generation and the irrelevance of the isotope integration step, have allowed the development of a highly optimized protocol that offers significant advantages over other published protocols: simplicity, no need for monitoring, minimal active time, high yields of labelled protein and cost reduction.

## Supplementary Information

Below is the link to the electronic supplementary material.

Supplementary file1 (DOCX 2353 KB)

## Data Availability

No datasets were generated or analysed during the current study.
