# Supplementary material for: Counterintuitive method improves yields of isotopically labelled proteins expressed in flask-cultured Escherichia coli
Source: J Biomol NMR. 2025 Mar 1;79(2):129–41. doi: 10.1007/s10858-025-00461-2 (PMC12078410; doi:10.1007/s10858-025-00461-2)
Supplement: Supplementary file 1 — Supplementary file1 (DOCX 2353 KB) [file 10858_2025_461_MOESM1_ESM.docx]

**SUPPLEMENTARY MATERIAL**

**Supplementary figure 1**: ^1^H-^13^C-HSQC spectra of sample obtained from 50 ml cultures with or without accommodation step previous to IPTG induction a. decoupled spectrum, b. coupled spectrum without accommodation step, c. coupled spectrum with one hour accommodation step previous to IPTG induction. The close similarity of panels b and c and the lack of a middle peak evince that the one hour accommodation step is not necessary and that the degree of ^13^C labeling is very high for both conditions as no triple peak (which would result from unlabelled sample) is seen in the coupled spectra.

**PROTOCOL**

"Unattended" Protocol for 13C-labelled protein expression.

- Day 1
  - Grow pre-inoculum (around 1ml in LB) at 30-37ºC overnight. This step can be eliminated if enough cells from colonies in a fresh plate or glycerol frozen cells are directly used.
- Day 2.
  - Prepare modified M9++ minimal medium (see table 1) and add **0.2% UNLABELED D-glucose** and 0.06% **LABELED** **^15^NH_4_Cl**.
  - Use an Tunair or Erlenmeyer flask with Volume ≥ 10-20 medium volume for the culture.
  - Inoculate with bacteria grown the day before to 0.05-0.1 OD_600_ (this is not too relevant).
  - Keep shaking vigorously (200rpm) at 25ºC for 24h.
- Day 3
  - OD_600_ should normally reach around 2-2.5 but it is not necessary to check.
  - Temper to the induction/expression temperature.
  - Add 1% **^13^C-D-glucose** and 0.3% **^15^NH_4_Cl** and IPTG according to your protocol/protein.

Accustoming the cells to the minimal medium or waiting for isotope incorporation is unnecessary.

- - Maintain culture as usual (20ºC or 25ºC, 24h ensure complete consumption of ^13^C and its incorporation to the protein)
- Day 3 or 4 (depending on expression conditions).
  - Harvest cells as usual

**RECIPES**

100mL modified M9++ minimal medium (modified from Cai et al (2019)).

To 80ml H_2_O add:

- LB medium 100 μL
- Trace elements 20 μL
- MgSO_4_ (1M) 100 μL
- 100 x BME vitamins 250 μL
- Thiamine (1mg/ml) 10 μL
- CaCl_2_ (1M) 20 μL
- 5xSalts M9++ 20 mL ALWAYS ADD AT THE END! (or at least after trace elements and CaCl_2_ to avoid precipitation) (salts recipe below)
- Antibiotic

Final concentration of each component:

- LB medium 1 ml/L
- FeSO_4_ ·7H_2_O 0.12 mg/L
- MnCl_2_ ·4H_2_O 0.024 mg/L
- CoCl_2_ ·6H_2_O 0.016 mg/L
- ZnSO_4_ ·7H_2_O 0.014 mg/L
- CuCl_2_ ·2H_2_O 0.006 mg/L
- H_3_BO_4_  0.0004 mg/L
- (NH_4_)_6_Mo_7_O_24_ ·4H_2_O 0.05 mg/L
- EDTA 100 mg/L
- MgSO_4_ 120.37mg/L
- BME vitamins 100x solution 2.5ml/L
- Thiamine 10mg/L
- CaCl_2_ (1M) 22.2mg/L
- 5xSalts M9++ 20 mL
- K_2_HPO_4_ 19 g/L
- KH_2_PO_4_ 5 g/L
- Na_2_HPO_4_ 9 g/L
- K_2_SO_4_ 2.4 g/L

Salts M9++ 5x

For 1 L:

- K_2_HPO_4_ 95 g
- KH_2_PO_4_ 25 g
- Na_2_HPO_4_ 45 g
- K_2_SO_4_ 12 g

Trace elements solution

For 1 L:

- FeSO_4_ ·7H_2_O 6.00 g
- MnCl_2_ ·4H_2_O 1.20 g
- CoCl_2_ ·6H_2_O 0.80 g
- ZnSO_4_ ·7H_2_O 0.70 g
- CuCl_2_ ·2H_2_O 0.30 g
- H_3_BO_4_  0.02 g
- (NH_4_)_6_Mo_7_O_24 ·_4H_2_O 0.25 g
- EDTA 5.00 g

LB medium

For 1 L:

- Yeast Extract 5 g
- Triptone 10 g
- NaCl 10 g
